# Effect of decay on detection of chronic wasting disease (CWD) in lymph nodes using enzyme-linked immunosorbent assay (ELISA)

**DOI:** 10.1080/19336896.2026.2727327

**Published:** 2026-09-02

**Authors:** Jameson Mori, Daniel Barr, Benjamin Johnson, Nelda Rivera, Peter Schlichting, Daniel Skinner, Jan Novakofski, Nohra Mateus-Pinilla

**Affiliations:** a Illinois Natural History Survey - Prairie Research Institute, University of Illinois Urbana-Champaign, Champaign, IL, USA; b Wisconsin Veterinary Diagnostic Laboratory, University of Wisconsin-Madison, Madison, WI, USA; c Illinois Department of Natural Resources, Division of Wildlife Resources, Springfield, IL, USA; d Department of Animal Sciences, University of Illinois Urbana-Champaign, Champaign, IL, USA; e Department of Pathobiology, University of Illinois Urbana-Champaign, Urbana, IL, USA; f Department of Natural Resources & Environmental Sciences, University of Illinois Urbana-Champaign, Urbana, IL, USA

**Keywords:** Cervid, chronic wasting disease, CWD, decay, decomposition

## Abstract

Surveillance of wildlife diseases poses multiple challenges, including the availability of samples for testing and, in some situations, issues with suboptimal tissue condition. Chronic wasting disease (CWD) is a prion disease threatening the health of wild and captive cervids that is diagnosed with enzyme-linked immunosorbent assay (ELISA) and/or immunohistochemistry (IHC) of retropharyngeal lymph nodes or obex tissue. IHC uses the three-dimensional structure from fresh preserved tissues to facilitate the detection of accumulated prion proteins PrP^CWD^ within the tissue architecture. ELISA does not require tissue structure and uses tissue lysates to detect PrP^CWD^, but its ability to detect PrP^CWD^ in degraded diagnostic samples is unknown. We thus examined how lymph node decomposition at 4°C and 27°C for up to 12 months affects the ability of ELISA to detect CWD prions in known CWD-positive lymph nodes from wild white-tailed deer (*Odocoileus virginianus*) in Illinois, USA. We found that prions remained detectable in 100% of the lymph nodes decayed for 12 months at both temperatures, though the detection percentage in each experimental group varied over time. Our findings are the first to demonstrate that USDA-APHIS approved ELISA assays can be used on extensively degraded lymph nodes for CWD surveillance, enabling testing of tissue in suboptimal conditions using.

## Introduction

Members of the family *Cervidae*, both wild and captive, are becoming increasingly affected by chronic wasting disease (CWD), a prion disease with high transmissibility and a 100% fatality rate [1–3]. First detected in the U.S. state of Colorado in the 1960s [4], it has since spread to 36 U.S. states and 4 Canadian provinces as of April 2025 [5]. A separate outbreak of CWD in Norway was first detected in 2016 and has since also been detected in Sweden and Finland [6]. South Korea has also documented cases of CWD in captive cervid farms traced back to imports from Canada [7].

CWD is a transmissible spongiform encephalopathy (TSE) caused by prions, which are a misfolded versions (PrP^CWD^) of anormal cell-surface proteins (PrP^c^) [8] that are most abundant in the nervous system and lymphatic tissues [9,10]. Prions are notably durable and resistant to proteases [11], dehydration [12] and a range of environmental conditions [13], requiring harsh treatments such as incineration at 1000°C [14] or the application of highly concentrated acids or bases [15] for complete deactivation. Though CWD is primarily transmitted through direct contact with an infected animal [16], the durability of the prions allows them to accumulate in the environment once shed by CWD-positive animals and cause indirect transmission via the ingestion or inhalation of contaminated soil [17], water [18] or plants [19,20] by naïve animals. Infected animals begin shedding prions early in the incubation period in their faeces [21,22], urine [22,23], saliva [22,23], blood [24,25], mucous [26], and other fluids and tissues, with this phenomenon becoming especially important in locations where cervids congregate such as mineral licks [27], deer scrapes [28] and feeders [29] where infected and susceptible deer share bodily fluids.

The high transmissibility of prions, 100% lethality and environmental stability make CWD both an immediate and long-term threat to cervid herd health. In addition to the consequences of shortened lifespans due to CWD infection and associated population declines [30,31], studies of the effects of maternal infection with CWD have found that the foetuses of CWD-positive female deer weigh less [32] and have smaller heads [33] than foetuses of CWD-negative females. Infected animals are also more likely to be involved in vehicle collisions [34], and the fawns of CWD-positive female deer are less likely to survive their first year of life [35]. For these reasons, the monitoring, controlling and managing of the spread of CWD is essential for reducing its detrimental impact on both captive and wild cervids.

Diagnostic testing is required to monitor the distribution and intensity of CWD, and a range of techniques for identifying prions are available. Histopathology of affected tissues using light or electron microscopes can be used to visually identify abnormal proteins and structures [36]. Antibody-based tests like Western blot, immunohistochemistry (IHC), enzyme-linked immunosorbent assay (ELISA), immunofluorescence assay and immunofluorometric assay use antibodies with fluorescent tags that bind to and illuminate pathogenic prions [36]. Flow cytometry can also be used to quantify prion presence, and *in vivo* bioassays can be conducted to see if inoculation of laboratory animals with diagnostic tissues causes disease [36]. Newer methods such as protein misfolding cyclic amplification (PMCA) and real-time quaking-induced conversion (RT-QuIC) amplify any abnormal prions present in the sample – similar in concept to polymerase chain reaction (PCR) techniques used for DNA – to enhance detection ability with the end diagnostic test, such as Western blot [36]. The selection of a test depends on multiple factors like the type and quality of the tissue sample, cost, desired turnaround time and required accuracy.

Ideally, diagnostic testing is conducted on fresh or frozen tissue samples with minimal autolysis, but situations do arise in which tissue decay has begun before the sample can be preserved. Though this can cause issues with test performance, past studies of antibody-based tests like Western blot [37,38], ELISA [37,39] and immunocytochemistry [40] have been shown to accurately detect BSE prions in bovine brain tissue at all stages of autolysis, including total liquification, and a study of scrapie in sheep and CWD in elk found that both ELISA and Western blot could detect prions in autolysed nervous and lymphoid tissues [41]. The duration of autolysis prior to diagnostic testing varied between these studies. Hayashi et al. (2004) found that both Western blot and ELISA could detect BSE prions after 4 days of decay at 37°C and Huang et al. (2011) determined that those same tests could also detect scrapie and CWD in tissues that had undergone autolysis for 15 days at 37°C. A study using immunocytochemistry was able to detect BSE in brainstems that had liquified for 80 days at either ambient conditions or 56°C [40]. Other studies testing buried cervid remains (burial duration unknown) were able to detect CWD prions using PMCA [42] and RT-QuIC [43], so it is known that prions persist and are detectable in tissues for months after the animal dies. Similarly, a study of skulls and brain tissue from BSE-affected cattle also detected infectious prions with PMCA after 5 years of decay [44].

The effects of a more extended period of autolysis are of interest because while CWD surveillance testing is done on samples collected from cervids killed (within the past 24 hours) during recreational hunting or culling efforts [45], there are instances where wildlife managers may have access to decomposing deer carcasses from which a lymph node or obex could be removed, like in the case of roadkill or outbreaks of epizootic haemorrhagic disease (EHD). If prion detection is possible with ELISA after longer decay times, this could expand the sample pool for CWD surveillance to include these convenient opportunistic samples, so long as the lymph node has some tissue that is intact enough to locate and remove. The success of these efforts does depend on several factors that impact both tissue as a whole and prion degradation specifically. In general, temperature, humidity/rain, sun exposure, insects and scavenging are the primary sources of variation in decomposition rates [46], with body mass also playing a role [47]. Prions can be degraded by some enzymes, oxidants, microbes and higher temperatures [48,49], with pH also playing a role in denaturing the pathogenic protein [50–52]. This emphasizes the need to consider and evaluate the interactions between environmental conditions and prion breakdown.

Though past studies have demonstrated that TSE prions can be identified even when tissues are in an advanced stage of decay, there are limitations to our current knowledge about testing autolysed tissues for CWD in particular, as well as other considerations that restrict testing options. At the present time, the United States Department of Agriculture (USDA) requires diagnostic testing for CWD to be done on retropharyngeal lymph nodes and/or obex tissue using IHC and/or ELISA [53–55], with some U.S. states like Illinois using ELISA for screening potential cases and IHC as confirmation [45]. IHC may not work with tissues that have lost their structure, but Huang et al. (2011) demonstrated that ELISA can detect CWD prions in autolyzed lymph nodes decayed for up to 15 days. Information is lacking, however, for longer time periods. To address this knowledge gap, our study examined if ELISA could detect CWD prions in known positive white-tailed deer retropharyngeal lymph nodes after open exposure to refrigeration at 4°C or incubation at 27°C for up to 12 months. All known positive lymph nodes tested positive again after a year of decay, demonstrating that ELISA can be used successfully on severely autolysed tissues.

## Methods

### White-tailed deer lymph nodes

As part of the CWD surveillance effort in Illinois, both retropharyngeal lymph nodes (hereafter referred to as ‘lymph nodes’) were collected from hunter-harvested and culled wild white-tailed deer (WTD) and sent to the Wisconsin Veterinary Diagnostic Laboratory (WVDL) for CWD testing. Samples were first screened with ELISA and compared to a threshold optical density to determine if the lymph node is considered an ‘initial reactor’ (likely positive) or ‘undetected’. This threshold was the mean of the negative controls for that ELISA well plate plus 0.035 and is typically between 0.045 and 0.048. All initial reactors were subsequently tested with IHC to confirm the CWD-positive diagnosis.

From the archive of WTD lymph nodes at the Wildlife Veterinary Epidemiology Laboratory (WVEL), 130 CWD-positive and 26 CWD-undetected lymph nodes were randomly selected and assigned unique identification numbers. Each lymph node was stored individually in a small plastic bag for the entire duration of the experiment. The initial optical densities (OD) of the CWD-positive lymph nodes ranged from 3.003 to 4, with a median and third quartile of 3.34 and 4, respectively. All the optical densities of the CWD-positive lymph nodes were much closer to the maximum ELISA output (4) than the minimum threshold for positivity (around 0.045). CWD-undetected lymph nodes had initial optical densities ranging from 0.013 to 0.028, with a median and third quartile both equal to 0.017, putting all negative controls well below the positive threshold.

It bears mentioning that serial testing with both ELISA and IHC to confirm a CWD positive case results in the complete destruction of that lymph node. This means that the CWD-positive lymph nodes used in this study were the second, untested lymph nodes from each animal, and so direct comparison between the original and final OD values should be avoided. Instead, what matters is the comparison between the final OD and the threshold for positivity.

### Experimental setup

**Figure 1.**
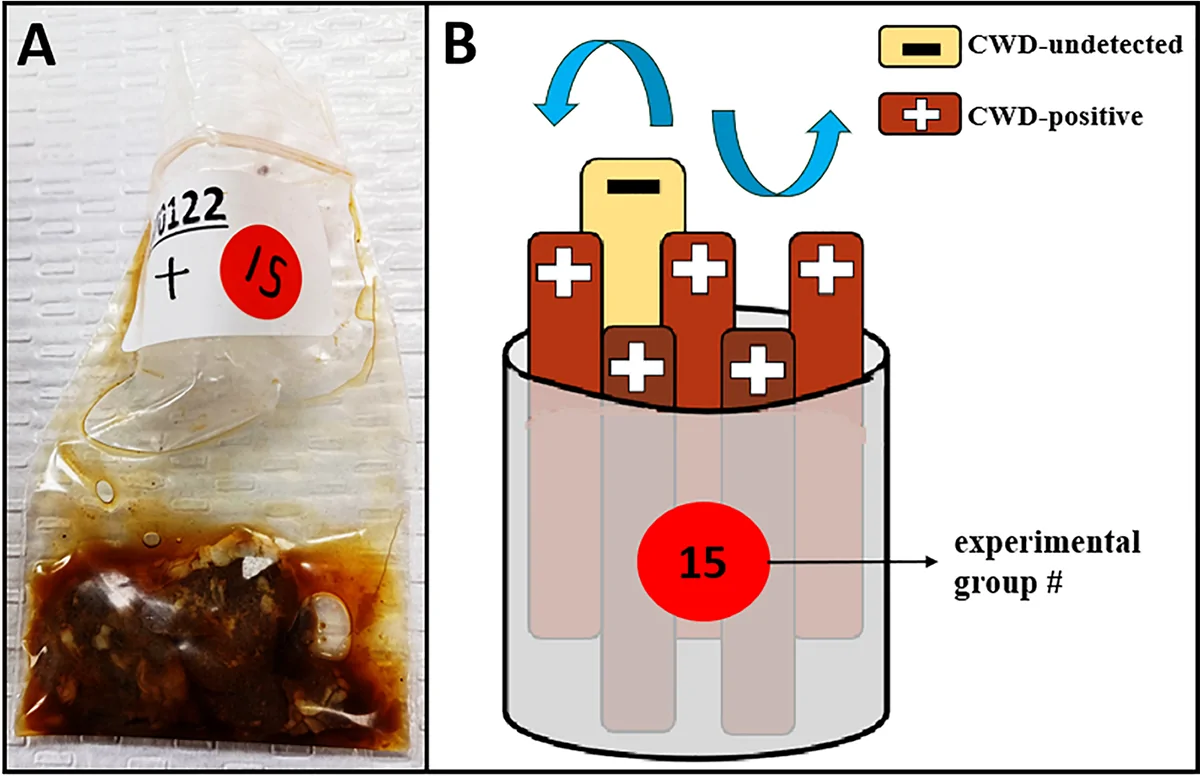
Example of the experimental group setup for the white-tailed deer (*Odocoileus virginianus*) lymph node decay experiment. (*A*) Shows a single lymph node sample bag, while (*B*) shows the setup of a group of samples in an individual experimental cup per group.

Lymph nodes were separated into 26 groups, each containing 1 CWD-undetected and 5 CWD-positive lymph nodes (*n = 6*). Lymph nodes were kept in the bags that they were received in, and each experimental group of 6 lymph nodes was labelled with its group number (1–26) (Figure 1(A)).

To expose the lymph nodes to the environment as they would be in a decomposing cervid carcass, the bags containing individual lymph nodes were left open and arranged in a plastic cup so that continuous air flow was allowed into each bag (Figure 1(B)). Each cup held all lymph nodes from a single experimental group (*n* = 6) and was labelled to reflect the group number (Figure 1(B)). This arrangement prevented contact between the lymph nodes and their fluids to ensure there was no cross-contamination either within or between experimental groups.

The impact of temperature on tissue decay was evaluated by storing groups 1–13 in a refrigerator at 4°C and groups 14–26 in an incubator at 27°C for the entire designated decay period. 4°C was chosen to approximate decay at a cold, but not frozen temperature such as would be experienced during the spring or fall in a temperate area like Illinois, while 27°C was chosen to approximate decay at a hotter temperature such as would be experienced during the summer.

### Lymph node decay schedule

To evaluate the detection of CWD prions in decaying CWD-positive lymph nodes over time, each of the 26 groups was exposed to a different treatment characterized by the combination of a particular temperature (4°C or 27°C) and decay time (0.5, 1, 2, 3, 4, 5, 6, 7, 8, 9, 10, 11 or 12 months). When a group reached its designated treatment duration, the individual lymph node bags were sealed, placed together in a larger plastic bag, and immediately frozen at −80°C [53,56] until all the lymph nodes were shipped frozen to the WVDL for retesting.

### Rehydration of desiccated lymph nodes

Lymph nodes that had become desiccated during the decay process required rehydration prior to conducting ELISA. A separate evaluation of the amount of water lost due to evaporation found that 59 of the 60 lymph nodes tested lost 65–81.2% of their weight when completely air dried. The lymph nodes used in this evaluation were not the same ones used in the decay experiment. This finding informed the addition to the ELISA protocol to soak desiccated lymph nodes in ~30 mL of reverse osmosis deionized water for 24–48 hours until rehydrated.

### Retesting of decayed lymph nodes for CWD

Once received by the WVDL, all lymph nodes were retested for CWD using a Bio-Rad ELISA kit following the procedure described below.

Decayed lymph node tissue (200 ± 20 mg) was homogenized in grinding tubes labelled with the sample identification number. To ensure proper homogenization, one large grinding bead was added to each tube. Tubes were closed firmly and placed in the PRECESS 48 homogenizer. Samples were homogenized on Mode 2 for 45 seconds at a speed setting of 6500 units ×2 cycles with a 30 second break between agitation cycles. Sample tubes were removed from the homogenizer and 400 mL of the sample was transferred to a specified well in a deep well plate. Unused homogenate was kept frozen at −20°C.

Selective purification of the CWD prion (PrP^CWD^) was achieved using an automated liquid handling instrument (NSP) and proteinase K treatment utilizing the Bio-Rad NSP software (v. 2.02) [57]. Selective concentration of PrP^CWD^ was achieved by centrifuging the sample at 4°C for 10 min at 2,000 relative centrifugal force. The supernatant was removed by a DW40 plate washer. Deep well plates were inverted onto a paper towel to remove all excess liquid from the sample. 25 µL of resolving buffer were added to each well, and deep well plates were incubated in a 100°C heating block for 5 min.

The Bio-Rad ELISA detection kit components were brought to room temperature for at least 30–60 min before use. The lyophilized positive control (a non-infectious recombinant prion protein) was resuspended in 4 mL of sample diluent. The negative control was phosphate-buffered saline (pH 7.4) supplemented with bovine serum albumin. 125 µL of sample diluent was added to all wells. Samples were thoroughly mixed by pipetting and transferred into the appropriate wells of the plate provided in the kit. 100 µL of the negative controls, positive controls and samples were added to their respective positions in plates according to the plate map. The plate was covered with sealing film and incubated on a 37°C microplate heating block for 30 min. During sample incubation, the 1x wash buffer (WB) was prepared by adding 100 mL of 10x WB to 900 mL of deionized reverse osmosis water. Once incubation was complete, unbound proteins were removed by washing the plate 3 times with WB using a Model 1575 Immunowash Microplate Washer. The conjugate was prepared by adding 1 mL of 10x conjugate to 9 mL of 1x WB. The contents of the tube were mixed by inversion and 100 µL of the contents were added to each well. The plate was covered with sealing film and incubated at 4°C for 30 min. After incubation, the plate was washed with WB five times using the Model 1575 Immunowash Microplate Washer. Before the end of the conjugate incubation, the chromagen solution was prepared by the addition of 1 mL of 10x chromagen to 10 mL substrate buffer. The tube was mixed by inversion, and 100 µL of chromagen solution was added to each well and incubated in the dark at room temperature for 30 min. After incubation, 100 µL of stop solution was added to each well.

Light absorbency of samples was measured in each well of the plate using an iMark Microplate Reader with 450-and 620 nm filters. Microplate Manager Software 6 (v. 6.3) [58] was used to analyse assay results. The software calculated the mean optical density (OD) (absorbance reading) of four negative controls and determined the cut-off value as the mean OD of the negative controls plus 0.035. Samples with OD values below the cut-off were classified as undetected; samples with OD values at or above the cut-off value were classified as positive. Test runs were considered valid only if the following conditions were met: (1) at least 3/4 of negative controls used for determining the cutoff value produced ODs < 0.150 and (2) at least 2/4 of the negative controls were < negative control OD * 1.4, and (3) both positive controls produced ODs > 1.

Once the ELISA results were obtained, lymph node identification numbers were linked to their original ELISA results, and the data was returned to the WVEL.

### Data analysis

Data on the OD and final CWD status of each lymph node were analysed to determine how long the CWD prions could be detected in decaying lymph nodes following treatments of different combinations of temperature (4°C or 27°C) and exposure duration (0.5 months or 1–12 months, in 1-month intervals).

### Statistical analysis

Two logistic regressions were conducted to determine whether the probability of a lymph node changing its CWD status to ‘undetected’ at the end of its decay period was impacted by temperature (*Equation 1*) or exposure duration (*Equation 2*).

glm(Change ~ Temperature) (*Equation 1*)

glm(Change ~ Exposure Duration) (*Equation 2*)

Temperature was treated as a categorical variable since it only had 2 possible values, and exposure duration was treated as continuous. A lymph node that tested CWD-positive at the beginning of the experiment but tested CWD-undetected at the end of its decay period was assigned the value ‘1’, while lymph nodes that remained CWD-positive were assigned the value ‘0’. Our dataset included 114 lymph nodes whose status remained CWD-positive and 10 whose status changed to CWD-undetected. A statistically significant regression coefficient for either covariate (*p*-value < 0.05) indicated that the covariate had an effect. Analysis was conducted in R [59] using the ‘lme4’ package [60].

## Results

### Experimental data output

Table 1 shows the ELISA testing results for the initially CWD-positive lymph nodes, with asterisks indicating the number of lymph nodes with an ‘error’ result (*n* = 6). Errors arose when the lymph nodes were rehydrated but were still too viscous and the homogenate clogged the automatic liquid handler’s pipette tips, preventing them from being able to be retested.

**Table 1.** Enzyme-linked immunosorbent assay (ELISA) results for initially CWD-positive white-tailed deer (*Odocoileus virginianus*) lymph nodes retested after the decay experiment. Lymph nodes underwent experimental environmental exposure in a refrigerator (4**°**C) or incubator (27**°**C) for the specified duration and were considered positive if their final optical density (OD) was the mean of the negative controls for that ELISA well plate plus 0.035, typically between 0.045 and 0.048. Each asterisk (*) indicates the number of lymph nodes that have an ‘error’ result (*n = 6*) within a group, which happened when lymph nodes were rehydrated but remained too viscous to be transferred with the laboratory’s instruments for further testing. Mean optical densities do not include lymph nodes with ‘error’ results.

Decay Duration (months)	Temperature 4°C				Temperature 27°C			
	Group #	Fraction Positive	Mean OD		Group #	Fraction Positive	Mean OD	
			Initial	Final			Initial	Final
0.5	1	5/5	3.261	3.352	14	5/5	3.886	2.43
1	2	5/5	3.269	2.223	15	5/5	4	2.532
2	3	4/5*	3.31	1.698	16	4/5*	3.275	1.092
3	4	3/5**	3.339	2.384	17	3/5**	3.1	0.121
4	5	5/5	3.292	1.784	18	4/5	3.324	0.806
5	6	5/5	3.119	0.753	19	2/5	3.208	0.406
6	7	5/5	3.183	1.193	20	4/5	3.397	0.273
7	8	5/5	3.268	0.905	21	5/5	3.887	0.825
8	9	4/5	3.242	0.76	22	3/5	4	0.981
9	10	5/5	3.163	1.82	23	5/5	4	1.414
10	11	5/5	3.289	0.352	24	3/5	4	0.226
11	12	5/5	3.419	1.674	25	5/5	4	0.295
12	13	5/5	3.273	1.689	26	5/5	3.17	0.149

The fraction of white-tailed deer lymph nodes in a group that remained positive for CWD prions after their designated decay duration varied over time (Table 1). The lowest positive fraction was group 19 (27°C for 5 months), which had only 2/5 lymph nodes test positive after their decay period (Table 1). Sixteen of the 26 groups had 5/5 lymph nodes test positive at the end of the experiment, 5 of the 26 had 4/5 test positive, and 4 of the 26 had 3/5 test positive (Table 1). The most notable finding is that all lymph nodes in groups that decayed for 12 months at both exposure temperatures (groups 12 and 26) still tested positive for CWD (Table 1). One negative control and six CWD-positive lymph nodes received an ‘error’ test result. All other CWD-undetected lymph nodes remained CWD-undetected, as shown in Table 2.

**Table 2.** Enzyme-linked immunosorbent assay (ELISA) results for initially CWD-undetected white-tailed deer (*Odocoileus virginianus*) lymph nodes (negative controls) retested after the decay experiment. Lymph nodes underwent experimental environmental exposure in a refrigerator (4**°**C) or incubator (27**°**C) for the specified duration and were considered undetected if their final optical density (OD) was less than the mean of the negative controls for that ELISA well plate plus 0.035, typically between 0.045 and 0.048. Each asterisk (*) indicates a lymph node that had an ‘error’ result (*n = 1*), which happened when lymph nodes were rehydrated but remained too viscous to be transferred with the laboratory’s instruments for further testing.

Decay Duration (months)	Temperature 4°C				Temperature 27°C			
	Group #	Fraction Undetected	OD		Group #	Fraction Undetected	OD	
			Initial	Final			Initial	Final
0.5	1	1	0.018	0.033	14	1	0.017	0.021
1	2	1	0.019	0.022	15	1	0.017	0.018
2	3	*	0.016	*	16	1	0.017	0.012
3	4	1	0.016	0.017	17	1	0.017	0.019
4	5	1	0.023	0.017	18	1	0.015	0.013
5	6	1	0.014	0.017	19	1	0.017	0.014
6	7	1	0.014	0.019	20	1	0.015	0.01
7	8	1	0.016	0.013	21	1	0.015	0.011
8	9	1	0.015	0.013	22	1	0.013	0.009
9	10	1	0.015	0.01	23	1	0.014	0.009
10	11	1	0.028	0.012	24	1	0.016	0.009
11	12	1	0.017	0.008	25	1	0.017	0.008
12	13	1	0.019	0.018	26	1	0.017	0.008

**Figure 2.**
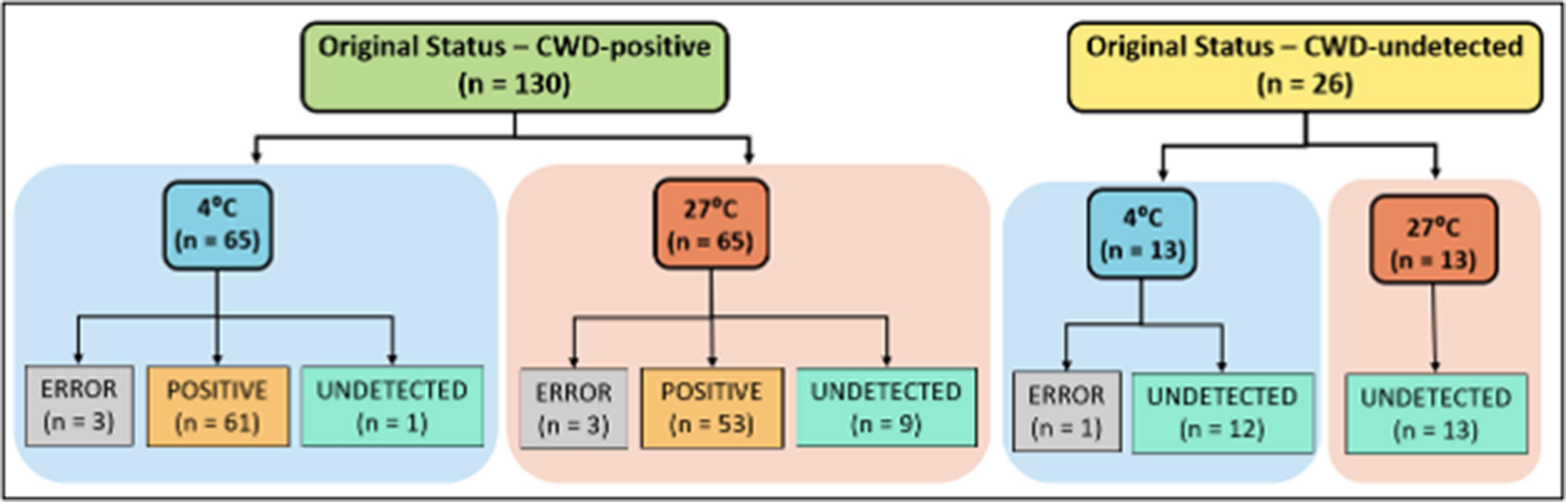
Flowchart of enzyme-linked immunosorbent assay (ELISA) results.

Figure 2 provides a breakdown of the ELISA test results for all tested lymph nodes. Only 10 out of 130 lymph nodes (7.7%) that were initially CWD-positive became CWD-undetected by the end of the study (Figure 2).

**Figure 3.**
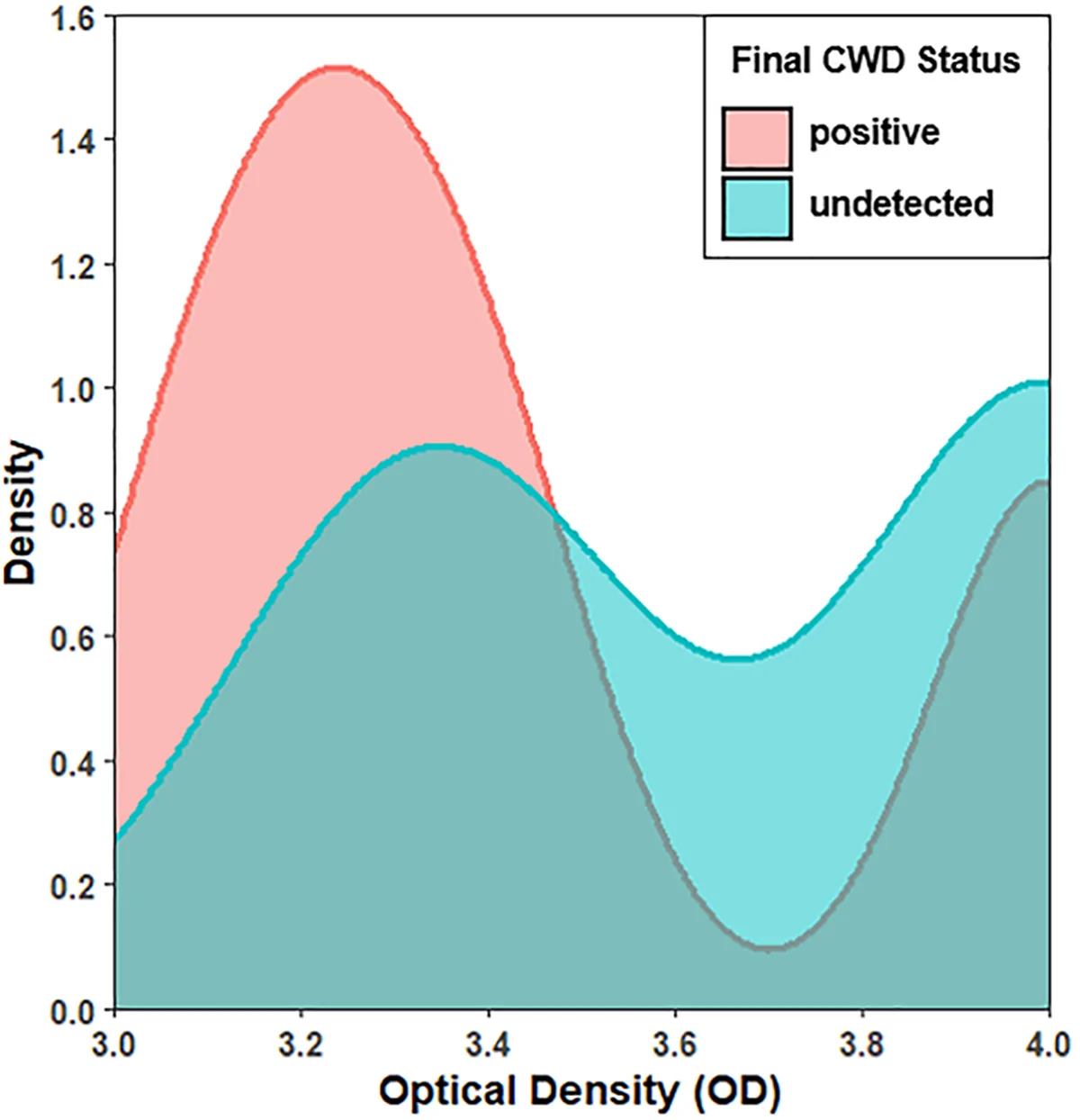
Density plot of CWD positive lymph nodes whose original optical densities (OD) were indicative of a positive CWD result and later received an “undetected” (blue) or “positive” (pink) result at the end of the experiment. Lymph nodes were deemed positive if their OD was greater than the mean of the negative controls for that ELISA well plate plus 0.035, typically between 0.045 and 0.048.

To determine if lymph nodes that were originally positive but then were undetected at the end of the study (blue; *n* = 10) had lower initial OD’s than the lymph nodes that tested positive throughout (pink; *n* = 114), we compared density plots of the original OD’s of the two groups (Figure 3).

There was a high degree of overlap between the original optical densities of lymph nodes that tested CWD-positive at the end of the experiment and those that tested CWD-undetected at the end. Based on this information, it was concluded that the change in ELISA status for lymph nodes that were CWD-undetected after decay was not due to those lymph nodes having a lower initial OD compared to the lymph nodes that remained CWD-positive. However, the sample size of lymph nodes that changed their test status was limited (*n* = 10) and was deemed insufficient for confirmation with statistical significance testing, and so this conclusion is based solely on visual inspection.

### Statistical analysis

The two logistic regressions relating temperature and exposure duration to the probability of a lymph node going from CWD-positive to CWD-undetected over the course of the experiment revealed that temperature had a statistically significant impact (*p*-value = 0.029), but exposure duration did not (*p*-value = 0.534). The regression coefficient for temperature was 2.338, meaning that lymph nodes decaying at 27°C were 936.05% or 10.36 times more likely to lose their CWD-positive status than lymph nodes decaying at 4°C. This supports the findings in Table 1 and Figure 2 that show more lymph nodes in the 27°C group became CWD-undetected than in the 4°C group.

## Discussion

This study of CWD detection in degraded white-tailed deer lymph nodes found that CWD prions were still detectable with ELISA after 12 months of decay, which has implications for prion contamination and persistence in soil [61–63], plants [19], water [18,64] or fomites (Table 1) [65,66]. The successful detection of CWD prions in these lymph nodes using ELISA expands upon the previous findings of Huang et al. (2011), who detected CWD in lymph nodes autolyzed for 15 days at 37°C, and also supports the findings of past studies that detected CWD or BSE prions in degraded tongues [42], nervous tissue [43,44], bone [43,44], connective tissue [43] and lymph nodes [43] using RT-QuIC and PMCA.

While all the lymph nodes that decayed for 11 and 12 months remained positive for both experimental temperatures, the percentage of initially positive tissues in each group that still tested positive after decay varied over time, particularly for the 27°C lymph nodes (Table 1). The lowest positive fraction observed for the groups stored at 27°C was 2/5 (40%), compared with the 4°C groups, which had a minimum of 4/5 (80%) (Table 1). Overall, there were more lymph nodes in the 27°C groups that lost their positive status (*n* = 9) than in the 4°C groups (*n* = 1) (Table 1). The logistic regression of temperature’s impact on ELISA status change reinforced this finding and provided additional evidence that higher temperatures increase the rate of pathogenic prion (PrP^CWD^) decay, with lymph nodes that decayed at 27°C being 10.34 times more likely to become CWD-undetected than those at 4°C. A similar phenomenon has been observed both in laboratory studies of the impact of heat from a thermocycler [67], or composting [14,68] on prion degradation. This emphasizes the importance of considering temperature when experimenting with prions and supports future efforts to collect more data on prion degradation at a wider range of temperatures, particularly temperatures that are normally experienced in the environment to better inform models of the role of prion contamination of soil, plant or water on transmission dynamics and disease [50]. It is interesting to note that for the lymph nodes tested, the duration of decay time did not have a significant effect on the loss of CWD-positive status (*p*-value = 0.534), reinforcing how resistant prions are to deactivation.

Prior studies have demonstrated that RT-QuIC [69] and PMCA [70] are more sensitive for prion detection than ELISA and produce fewer false negatives [69]. This means that prion levels may decline to a point where ELISA cannot detect them even if they are present, though whether those prion levels would be able to cause infection is unknown. The challenge of detecting prions at low concentrations with ELISA is the biggest limitation of using this test; however, the overall success of detecting CWD prions in the diagnostic lymph node samples over time, even with advanced tissue decay, underscores the strength of ELISA as a diagnostic tool for CWD when IHC cannot be performed due to tissue structural breakdown [71]. ELISA also has the advantage of being a faster, less expensive test with high throughput, making it more suitable for wildlife disease surveillance [72], though testing is only possible if the person collecting the tissue can locate and remove the degraded lymph node(s), which might not be possible depending on the condition of the carcass.

In the case of lymph node deterioration to the point where isolation is difficult, alternative approaches would be necessary. Targeting the obex instead of the lymph nodes is one option, as the obex is the other gold standard diagnostic tissue for CWD testing [53–55]. It would be worthwhile to study the rate of decay in the obex relative to the lymph nodes and how that decay impacts CWD ELISA results, as well as investigate the potential for using other body tissues for diagnosis. Possible options include bone marrow and non-obex brain tissue, both of which were found to be CWD-positive in decomposed deer carcasses using RT-QuIC [43]. It would also be beneficial to develop guidelines for locating and collecting lymph nodes from decayed carcasses.

The success of CWD detection in this experiment supports expanding the sources of samples that could be used to collect lymph nodes to be submitted for CWD diagnostic testing to include older samples with decreased tissue integrity, such as older roadkill [73–75] and samples from deer that may have died during an EHD outbreak. This could increase wildlife managers’ ability to evaluate disease burden in wild cervid herds from sources that may be more convenient or available at times other than during the hunting season or culling efforts.

In total, there were 7 lymph nodes (6 CWD-positive and 1 negative control) that were not successfully tested, all of which were in the 2- (groups 2 and 16) or 3-month groups (groups 3 and 17) (Table 1). That all 7 lymph nodes belonged to only the 2- or 3-month groups could be random chance, or it could be indicative of potential issues with the experimental setup or preservation process. In light of these results, we recommend that future studies run multiple negative controls per group to reduce the impact of errors.

The main limitation of this study is that temperature was the only environmental characteristic explicitly evaluated. Future efforts should explore the effects of other environmental conditions on tissue decay and CWD detection, such as composting [14], soil characteristics [76,77] and freeze–thaw cycling [12,21], as well as the potential impacts of CWD strain and the cervid’s prion protein haplotype. Additional investigation into the impact on tissue degradation in environmental conditions more similar to those that would be experienced by deer in the wild would improve the direct applicability of these findings to real-world situations and better inform sampling guidelines. It would also be informative to compare ELISA with RT-QuIC and PMCA to generate a timeline of detectability with each diagnostic test. Lastly, due to the nature of disease surveillance for wildlife, we had no knowledge of how long each deer had been infected, which influences the amount of prion accumulation in the lymph nodes. It may also be of interest to examine ELISA results for lymph nodes decaying in conditions that do not lead to desiccation, such as within a sealed plastic bag to more fully investigate the impact of tissue breakdown on laboratory outcomes. In future studies in which desiccation is a risk, we also would recommend measuring the starting weight of each lymph node to more precisely determine water loss and the amount of water to add to the tissue prior to conducting ELISA.

Overall, however, this study was able to determine, for the first time, that ELISA may still be used on degraded lymph nodes even after a year of decay, opening up possibility of expanding diagnostic testing to previously excluded animals.

## Conclusion

Our study confirms that CWD prions remain detectable in cervid lymph nodes via ELISA for up to 12 months of environmental exposure. We also found that warmer temperatures lead to greater fluctuations in positivity, likely due to their effects on prion degradation. This study emphasizes the value and effectiveness of ELISA as a diagnostic tool, especially for tissues that have lost architectural integrity.

## Data Availability

All data generated in this study are available at the Illinois Databank: https://databank.illinois.edu/datasets/IDB-9298512?code=buOPNurMf686UuaQTvkFTrFsDSeTFd0DV5RFOTjIqRw.
